# Biomechanical effects of different vertebral heights after augmentation of osteoporotic vertebral compression fracture: a three-dimensional finite element analysis

**DOI:** 10.1186/s13018-018-0733-1

**Published:** 2018-02-08

**Authors:** Wen-Tao Zhao, Da-Ping Qin, Xiao-Gang Zhang, Zhi-Peng Wang, Zun Tong

**Affiliations:** 1 Gansu University of Chinese Medicine, No. 35, Dingxi East Rd., Chengguan District, Lanzhou, 730000 Gansu Province People’s Republic of China; 2 Affiliated Hospital of Gansu University of Chinese Medicine, No. 735, Jiayuguan West Rd., Chengguan District, Lanzhou, 730000 Gansu Province People’s Republic of China; 3 Yunnan University of Traditional Chinese Medicine, No. 1076, Yuhua Rd., Chenggong District, Kunming, 650500 Yunnan Province People’s Republic of China

**Keywords:** Vertebral augmentation, Von Mises stress, Osteoporotic vertebral compression fracture

## Abstract

**Background:**

Clinical results have shown that different vertebral heights have been restored post-augmentation of osteoporotic vertebral compression fractures (OVCFs) and the treatment results are consistent. However, no significant results regarding biomechanical effects post-augmentation have been found with different types of vertebral deformity or vertebral heights by biomechanical analysis. Therefore, the present study aimed to investigate the biomechanical effects between different vertebral heights of OVCFs before and after augmentation using three-dimensional finite element analysis.

**Methods:**

Four patients with OVCFs of T12 underwent computed tomography (CT) of the T11-L1 levels. The CT images were reconstructed as simulated three-dimensional finite-element models of the T11-L1 levels (before and after the T12 vertebra was augmented with cement). Four different kinds of vertebral height models included Genant semi-quantitative grades 0, 1, 2, and 3, which simulated unilateral augmentation. These models were assumed to represent vertical compression and flexion, left flexion, and right flexion loads, and the von Mises stresses of the T12 vertebral body were assessed under different vertebral heights before and after bone cement augmentation.

**Results:**

Data showed that the von Mises stresses significantly increased under four loads of OVCFs of the T12 vertebral body before the operation from grade 0 to grade 3 vertebral heights. The maximum stress of grade 3 vertebral height pre-augmentation was produced at approximately 200%, and at more than 200% for grade 0. The von Mises stresses were significantly different between different vertebral heights preoperatively. The von Mises stresses of the T12 vertebral body significantly decreased in four different loads and at different vertebral body heights (grades 0–3) after augmentation. There was no significant difference between the von Mises stresses of grade 0, 1, and 3 vertebral heights postoperatively. The von Mises stress significantly decreased between pre-augmentation and post-augmentation in T12 OVCF models of grade 0–3 vertebral heights.

**Conclusion:**

Vertebral augmentation can sufficiently reduce von Mises stresses at different heights of OVCFs of the vertebral body, although this technique does not completely restore vertebral height to the anatomical criteria.

## Background

Osteoporotic vertebral compression fracture (OVCF) is a common disease in the elderly population accompanied by decreased bone mineral density [1], which can cause acute or chronic back pain, functional limitations of the spine, a thoracolumbar vertebral deformity, vertebral height (VH) loss, and deterioration of quality of life [2, 3]. OVCFs occur more frequently than other osteoporotic fractures, such as hip fractures and distal radius fractures [4], and they have become a more increasingly serious disease and a significant health problem worldwide that will obviously increase economic burden to society and family in the future [5].

Before vertebral augmentation (VA) techniques were developed, conservative treatment options of OVCFs included bed rest, analgesic drugs, calcium supplementation, antiresorptive drugs, and a spine brace for a few weeks. To stabilize fractures quickly, relieve pain fast, and achieve restoration of VH for the treatment of OVCFs, two minimally invasive VA procedures, percutaneous vertebroplasty (PVP) and percutaneous kyphoplasty (PKP), have been performed by percutaneously injecting bone cement into the fractured vertebral body [6, 7]. The difference between these procedures is that PVP is used to stabilize the fracture primarily through a very small skin incision in which bone cement is injected, and PKP can achieve more restoration of VH through insertion of a balloon through the same skin incision and expansion of the fractured vertebral body before the cement injection [8]. Two kinds of bone cement injection methods are used in PKP and PVP: unilateral or bilateral injection. The benefit of these minimally invasive procedures compared to conservative treatment or open surgery is better pain relief and functional spine improvement [4]. When bone cement is injected into the vertebral body, it may have analgesic effects by consolidating micro-fractures and reducing the mechanical stress generated by body weight and with activity, and it may destroy bone nerve endings by a cytotoxic and exothermal action in the course of cement polymerization [9].

Biomechanical tests of PKP have confirmed that cement augmentation improved vertebral fracture stability, and there were no significant differences in VH restoration in in vitro studies [10, 11]. The results of restoration of mechanical stability through VA have been also confirmed by finite element analysis (FEA), which can predict the long-term stability of a bone after cement augmentation through reliable models [8, 12].

Many previous studies have reported excellent clinical results of augmentation with PVP and PKP, which strengthen augmented vertebral bodies and can result in significant, rapid pain relief in 80–90% of patients, stabilize vertebral compression fractures quickly, and improve spinal deformity [1–3, 5]. According to clinical measurements on vertebral compression fractures treated with PVP or PKP, a certain amount of height restoration is achieved only in 66% of treated patients [8]. VH and kyphosis deformities can result in different recovery and correction, but pain and function can be adequately improved. Many surgeons think that the treatment of fractures requires good reduction, as the shift in the fracture segment may affect healing and function; however, several prior analyses have focused on total pain relief, quality of life, and safety outcomes and did not observe the effects between the different heights of the vertebral body after VA [13–15]. Therefore, it is necessary to explore the effect of vertebral strength on different VHs. Although there are many ways to classify OVCFs, the distinction between changes of VH is a more intuitive way to classify these fractures. Clinical results have shown that different VHs have been restored postoperatively and the treatment results are consistent. Currently, no significant results regarding biomechanical effects post-augmentation have been reported with different VHs by biomechanical analysis. FEA is likely to be the gold standard as an alternative to bone strength research methods [16], and it may help assess vertebral fracture stability as a biomechanical analysis [17]. The aim of the present study was to explore the biomechanical effects between different VHs of OVCFs before and after augmentation by three-dimensional (3D) FEA. By analyzing the differences in stress changes, we will determine the consistency of biomechanical results and clinical findings for deciding whether good reduction must be performed through surgery.

## Methods

This study was granted an exemption from requiring ethics approval by the ethics committee of the Affiliated Hospital of Gansu University of Chinese Medicine. The authors obtained patient consent before enrolling participants in this study.

### Patients of the FEA models of T12 OVCFs

**Fig. 1:**
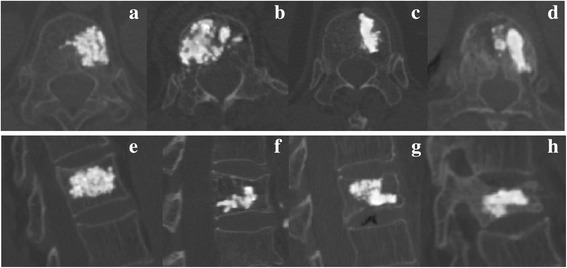
Computed tomography images of 4 patients with T12 osteoporotic vertebral compression fractures after vertebral augmentation. **a**–**d** Cross-section images of grade 0–3 vertebral height; **e**–**h** sagittal images of grade 0–3 vertebral height

In this study, four female volunteers with T12 OVCFs underwent computed tomography (CT) from the T11 level to the L1 level after VA, with a slice thickness of 0.625 mm. The CT images were reconstructed to simulate 3D FEA models. Inclusion criteria of OVCFs were a fracture due to acute minor or mild external trauma, individuals with a visual analog scale (VAS) score for back pain ≥ 5, the affected vertebral body showed a hypointense signal on T1-weighted magnetic resonance imaging scans and hyperintense signal on T2-weighted magnetic resonance imaging scans, and those with a *T* score ≤ − 1.5 for bone mineral density detected by dual-energy X-ray absorptiometry. The exclusion criterion was individuals with other pathological fractures detected by radiography. VHs of postoperative CT images were evaluated by Genant semi-quantitative grades 0–3. Vertebral bodies were graded as normal (grade 0), mildly deformed (grade 1, approximately 20–25% reduction anteriorly), moderately deformed (grade 2, approximately 25–40% reduction in any height and a reduction in area of 20–40%), and severely deformed (grade 3, approximately 40% reduction in any height and area) [18]. Cross-section images of the four different grades are shown in Fig. 1a–h. All CT images were saved in Digital Imaging and Communications in Medicine (DICOM) format.

### The construction of T11-L1 3D models and T12 fracture models

Four 3D models were developed based on the CT scans of T11-L1 vertebral bodies. The CT images of 4 patients in DICOM format were imported into Materialise Interactive Medical Image Control System (Mimics, version 10.01; Materialise, Inc., Leuven, Belgium) to generate the 3D model of the T11-T12-L1 vertebral bodies, including the cortical (1 mm thick) and cancellous bone. The following steps from the segmentation menu were performed: threshold segmentation was used to separate the bone and soft tissue, and editing mask tools were used to edit the image shape, select the desired area, fill the image area appearing in the gap, and split out the required contour layer by layer. Finally, 3D models were reconstructed through the edit mask option. The reconstructed 3D models were saved in STL format.

**Fig. 2:**
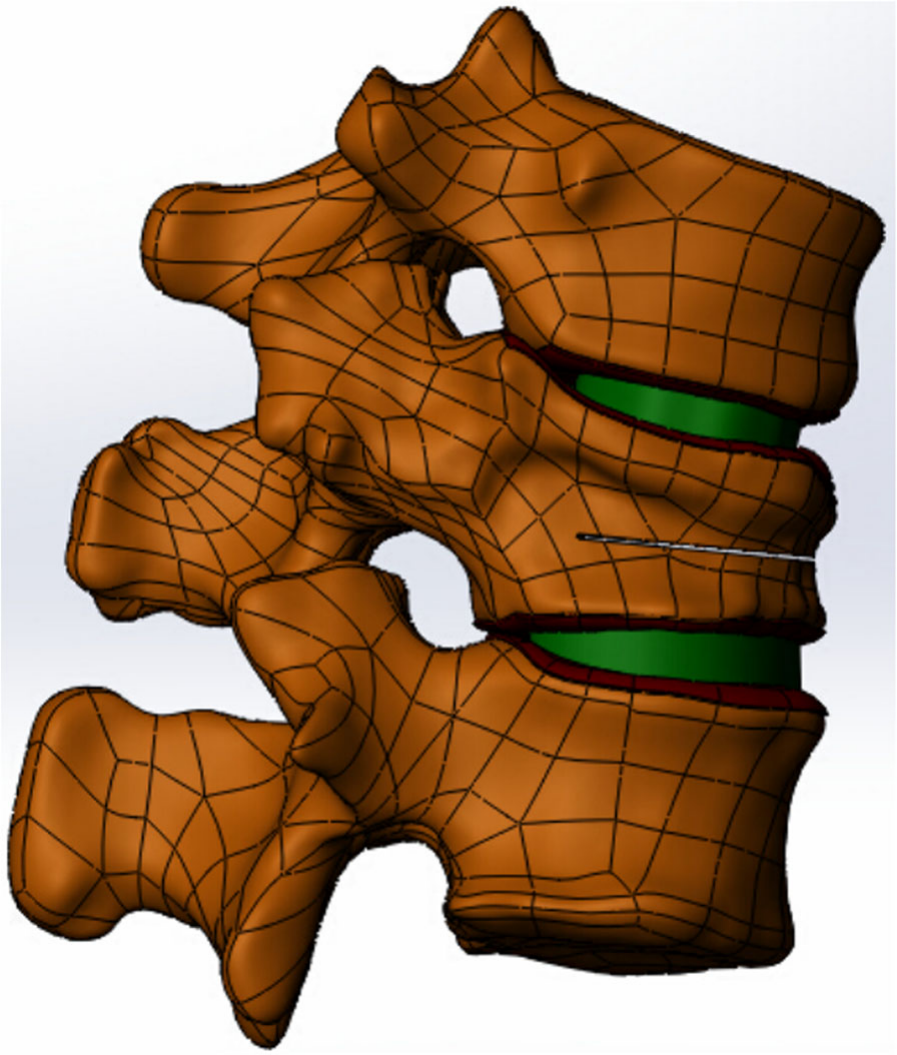
T11-L1 three-dimensional model and T12 fracture model

STL models were imported into automatic reverse engineering software, Geomagic studio (version 2012; Geomagic, Raindrop Geomagic, Research Triangle Park, NC, USA) for smooth noise reduction, feature removal, and structural patches and fitting surfaces. Then, the surface of the 3D models of the vertebral bodies were generated and saved in STP format. The models were optimized using Geomagic studio software and imported into Solidworks (version 2012; Dassault Systems, SolidWorks Corp., Santa Monica, CA), where T11, T12, L1 vertebral bodies were assembled for transposition and the surface command was used to generate the end-plate, cartilage, and intervertebral disc (nucleus pulposus and annulus). The desired 3D model was obtained by assembling these structures. Using a previously reported simulation method, models of the T12 fracture line were produced using the surface command to cut the vertebral body to produce the 0.5-mm fracture line (Fig. 2) [19].

### The construction of T12 models post-augmentation

**Fig. 3:**
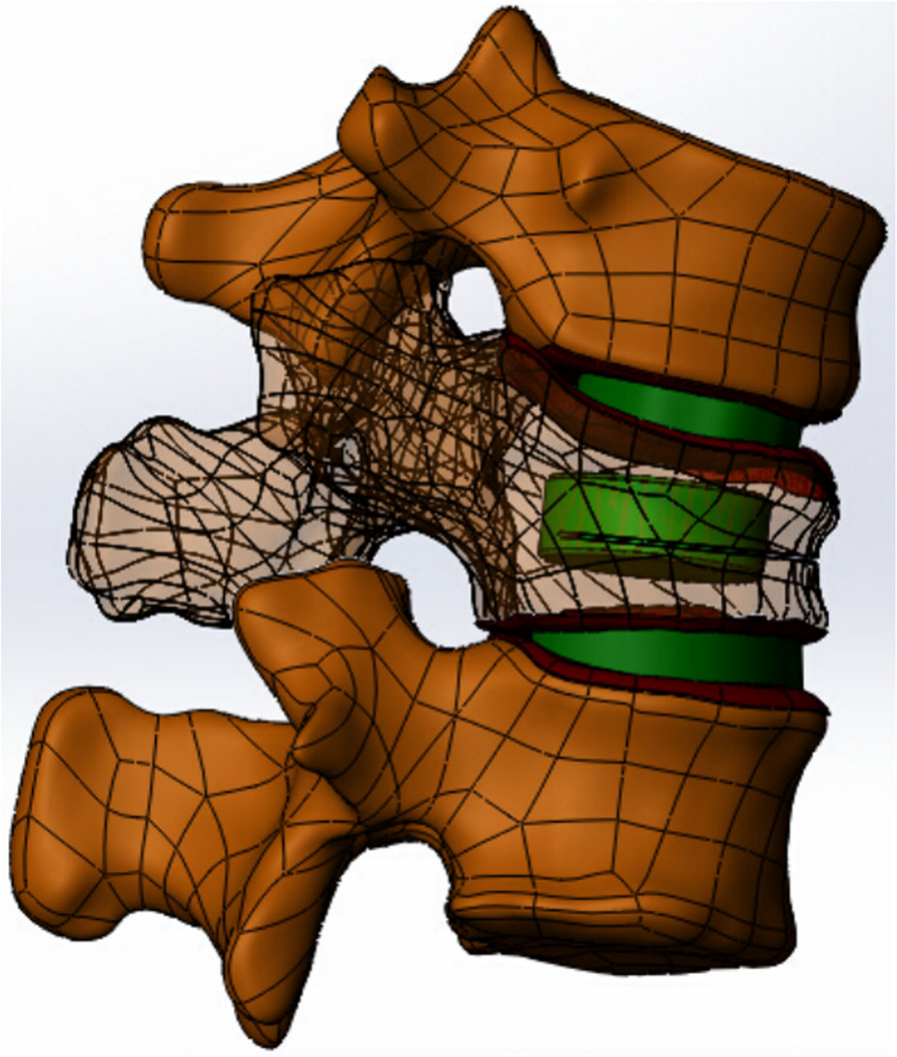
T11-L1 three-dimensional model and the T12 vertebral body after augmentation

STP models were imported into Solidworks software. Unilateral bone cement injection models were produced by software; a 4-mL upright pillar similar to a bone cement model was used; the bone cement in the T12 vertebral model was assembled in the center using the assembly command; and then through the software’s Boolean operation function, excess bones were removed, and the bone cement model was assembled into the vertebral body (Fig. 3). The 3D model of the T12 vertebral body after bone cement augmentation was obtained. Finally, the various models that included the T12-L1 vertebral bodies, bone cement, intervertebral discs, end-plates, and cartilages were assembled by the software to generate integral 3D parts.

### FEA models

The material properties used in recent studies about OVCFs are shown in Table 1 [20–23]. FEA was performed using ANSYS software (version 17.0, ANSYS, Canonsburg, PA, USA). The 3D models, including the cortical and cancellous bone cement, end-plate, cartilage, and intervertebral disc (nucleus pulposus and annulus), were imported into ANSYS software.

**Table 1:** Material properties of finite element analysis models

Component	Young modulus (MPa)	Poisson ratio
Cortical bone	8040 (67% normal)	0.3
Cancellous bone	34 (34% normal)	0.25
Bony end-plate	670 (67% normal)	0.4
Annulus	4.2	0.45
Nucleus pulposus	1	0.4999
Cartilage	10	0.4
Cement	3000	0.4
ALL	20	0.3
PLL	70	0.3
ISL	28	0.3
SSL	28	0.3
LF	50	0.3
CL	26	0.3

*ALL* anterior longitudinal ligament, *PLL* posterior longitudinal ligament, *ISL* interspinous ligament, *SSL* supraspinal ligament, *LF* ligamentum flavum, *CL* capsular ligament

**Fig. 4:**
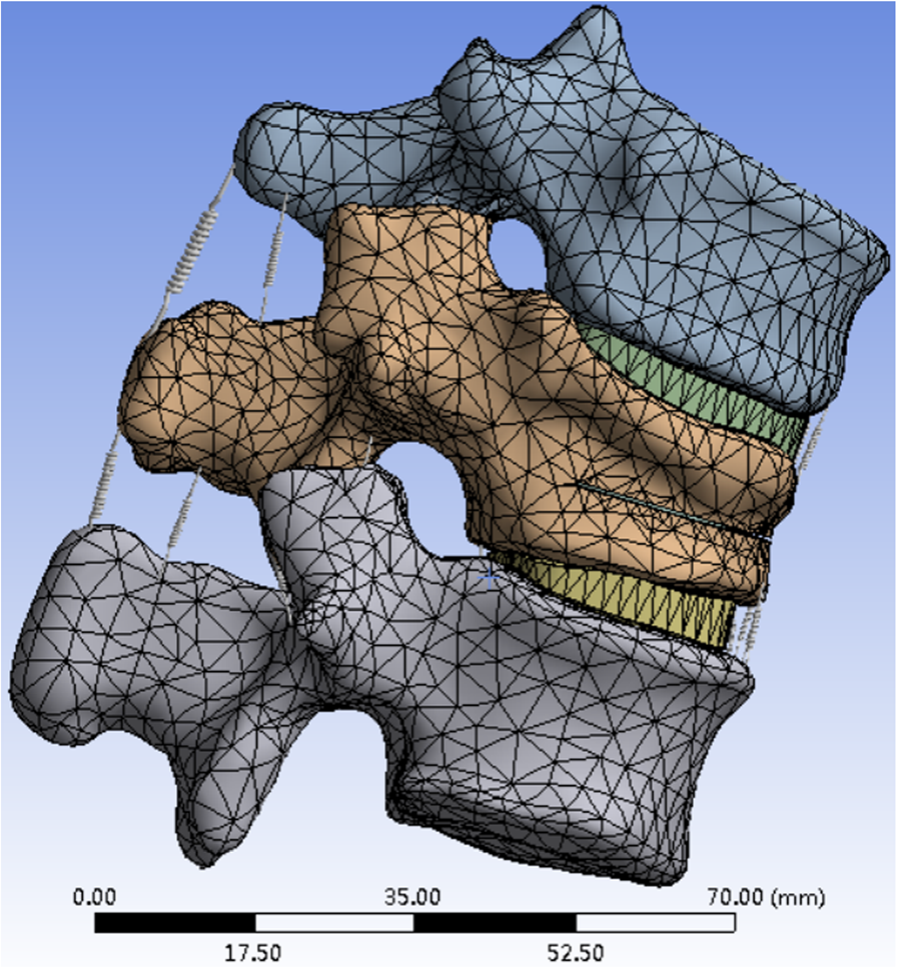
T11-L1 finite element analysis model

Supplemental components included the anterior longitudinal ligament (ALL), posterior longitudinal ligament (PLL), interspinous ligament (ISL), supraspinal ligament (SSL), ligamentum flavum (LF), and capsular ligament (CL). All models and bone cement were assigned linear elastic isotropic material properties. The element types of cortical bone, cancellous bone, bony end-plate, facet joint cartilage, and nucleus pulposus were defined as solid elements with a material representation of linear isotropic elasticity. The element types of the ALL, PLL, ISL, SSL, CL, and LF enable tension deformation without compression behavior. The end-plate, cartilage, and intervertebral disc (nucleus pulposus and annulus) were divided into 2-mm mesh. Cortical and cancellous bone cement were divided into 5-mm mesh. The mesh, nodes, and units are self-generated by the software. Connections between the end-plate and vertebral body, end-plate and intervertebral disc, and cartilage and bones were bonded. Connections between the cartilage and cartilage were frictionless. The lower edge of the L1 vertebral was set to fixed. The loads of vertical compression, flexion, and right lateral bending, or left lateral bending with four different loads were separately applied from the T11 vertebral upper edge. The pre-process for FEA was performed using meshed models (i.e., a meshing element was used, and material properties and boundary conditions were applied) (Fig. 4).

### FEA

Static FEA was performed by simulating the different extent of the fracture using four grades of VH of the T12 OVCF and two kinds of models: fresh OVCF (pre-augmentation) and OVCF after cement augmentation (post-augmentation). Five hundred newtons for vertical compression load and 7.5 N.m moment were applied for vertical compression and flexion, left flexion, and right flexion in all models. According to the spinal three-column concept, the load and moment were applied to the superior end-plate and articular facets of the T11 vertical body, with 85% of these on the anterior-middle column and 15% on the posterior column [19, 24–26]. Since the aim of this study was to evaluate the overall biomechanical changes of the vertebral body, the overall von Mises stresses on the T12 vertical bodies were calculated to evaluate the effects of cement augmentation. These FEA models of the T11-L1 vertebral bodies under vertical flexion and right or left flexion load were verified similarly to those published in the literature [27–29]. The normal model was validated according to published FEA models of human cadaveric thoracolumbar spines by ANSYS [23, 29]. Results of stress on the T12 vertebral body and stress cloud images at the end of the analysis can be exported to a computer. However, in this study, images of the T12 vertebral body and upper end-plate under stress are shown because the loads were conducted from the top to the bottom.

### Statistical analysis

The von Mises stresses on T12 vertebral bodies pre-augmentation and post-augmentation were applied under vertical compression and flexion, left flexion, and right flexion loads. One-way analysis of variance was used to analyze the effect of different loads and VHs before and after augmentation. SPSS software (version 17.0, IBM Corp., Armonk, NY, USA) was used to perform all statistical analyses. *P* < 0.05 was considered statistically significant.

## Results

Participants’ ages were 63, 71, 77, and 84 years, and VAS scores were 6, 8, 7, and 6, respectively. All patients underwent unilateral PKP. VAS scores decreased to 2, 2, 2, and 1 on the second day after surgery.

### Stress on the T12 vertebral body for different VHs pre-augmentation

**Fig. 5:**
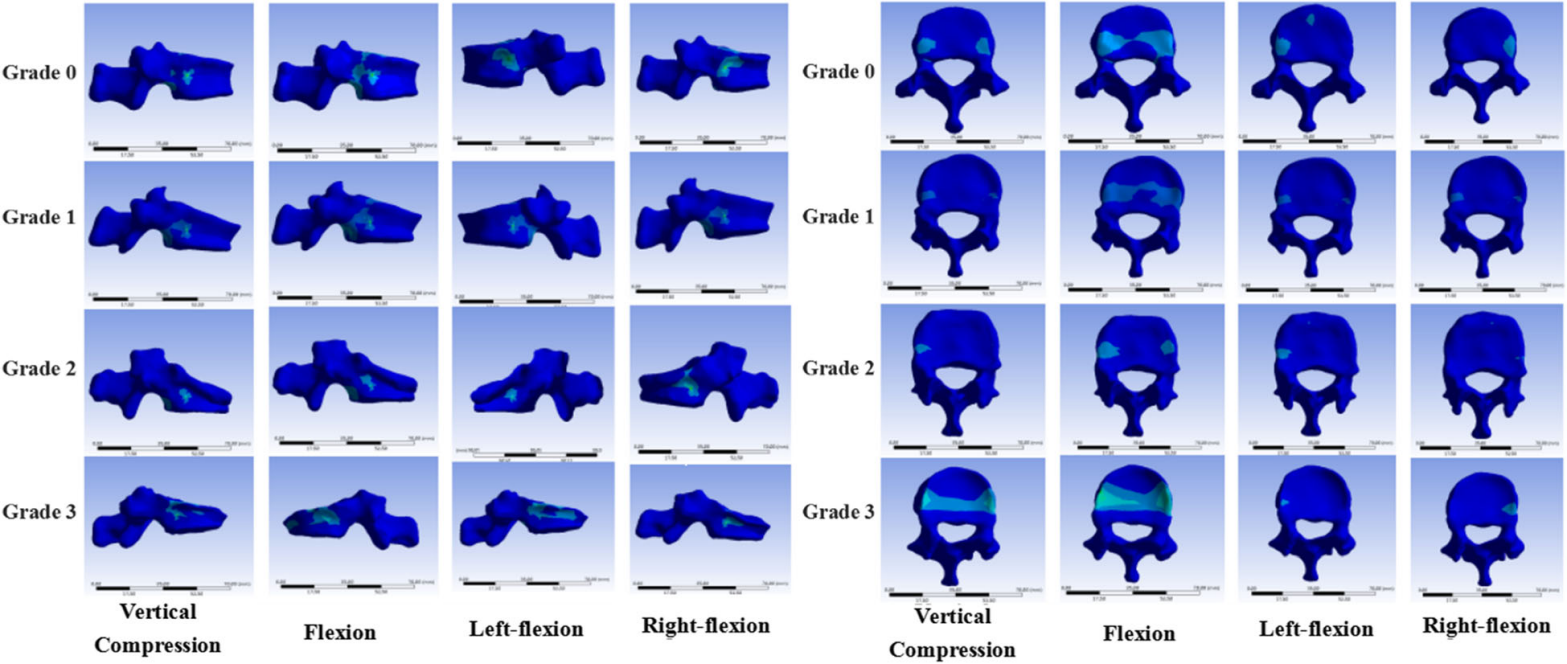
Nephograms of the von Mises stresses on fresh T12 osteoporotic vertebral compression fractures. The side and upper end-plate are shown

**Fig. 6:**
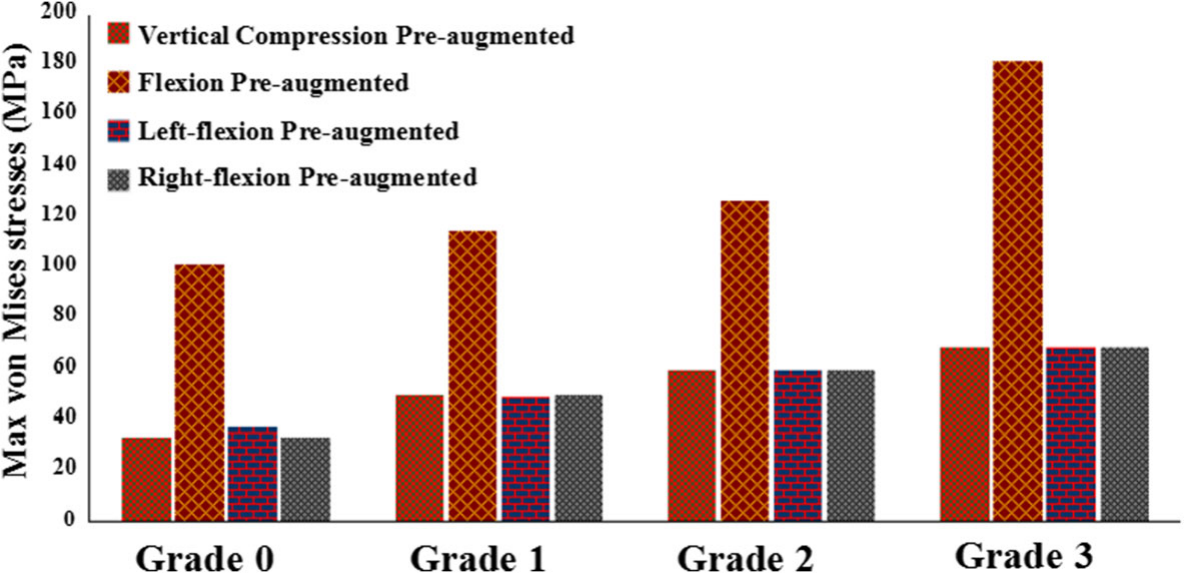
The von Mises stresses on fresh T12 osteoporotic vertebral compression fractures

Results were analyzed by four models of T12 OVCFs with grade 0–3 VHs before augmentation. The von Mises stresses of different VHs of T12 OVCFs under vertical compression and flexion, left flexion, and right flexion are shown in Figs. 5 and 6. Under vertical compression load, von Mises stresses on the T12 vertebral body for grades 0, 1, 2, and 3 were 33.282, 49.84, 59.93, and 68.966 MPa, respectively. Similarly, the von Mises stresses increased under flexion load from 101.89 MPa to 181.93 MPa. Regarding the different VHs, stresses of fresh OVCFs of the T12 vertebral body had a similar trend of change, which was that more height loss increased stress. The maximum stress of grade 3 VH pre-augmentation was produced at approximately 200% in flexion and left flexion, and at more than 200% for grade 0 in vertical compression and right flexion. There were significant differences in the loads between different VHs preoperatively.

### Stress on the T12 vertebral body for different VHs post-augmentation

**Fig. 7:**
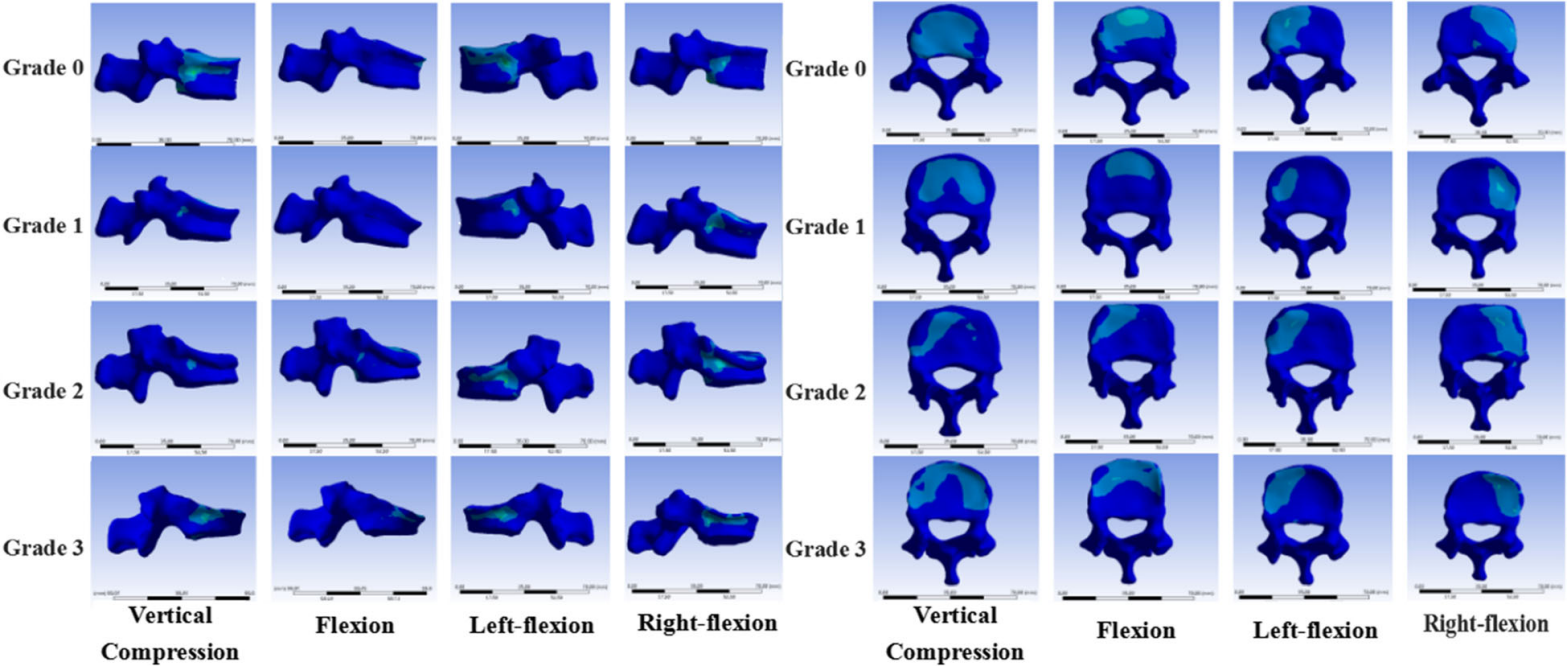
Nephograms of the von Mises stresses on T12 osteoporotic vertebral compression fractures after augmentation. The side and upper end-plate are shown

**Fig. 8:**
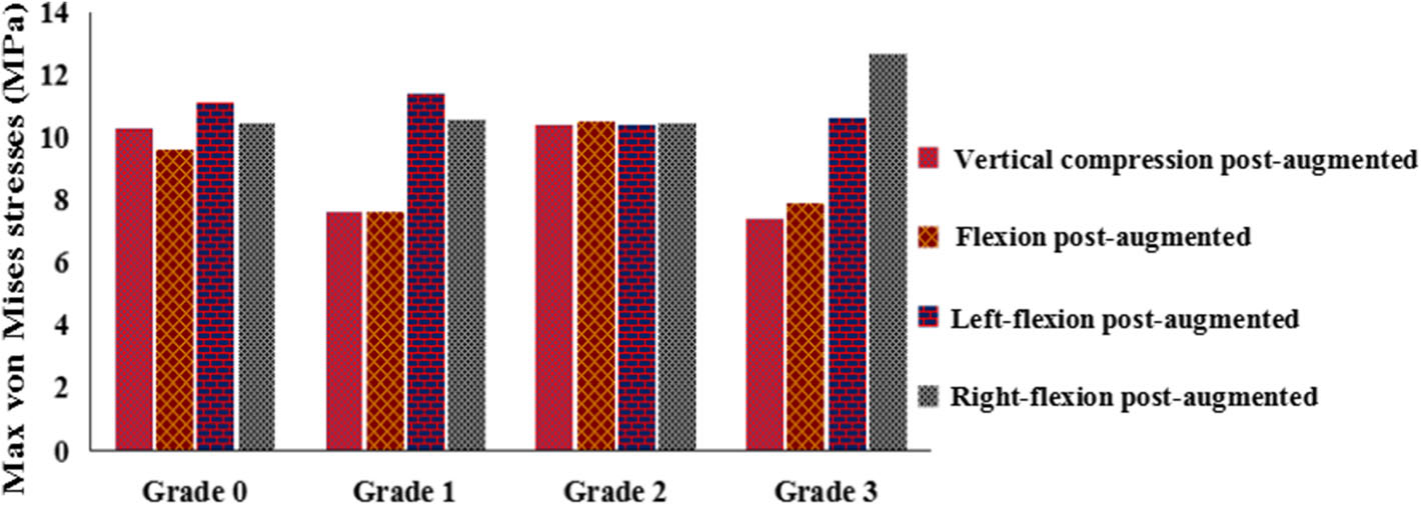
The von Mises stresses on the T12 vertebral body after augmentation

The von Mises stresses of different VHs post-augmentation of the T12 vertebral body under vertical compression and flexion, left flexion, and right flexion are shown in Figs. 7 and 8. Augmentation significantly decreased the stress on the T12 vertebral body in all four different loads for grade 0–3 VH. The maximum stress under flexion was 10.505 MPa for grade 3 VH. The maximum stress under all loads was 10.575 MPa in flexion for grade 1 VH. A comparison of postoperative loads among the four grades of VH showed that there was a significant difference between grades 0, 1, and 3 and grade 2, and no significant difference between grade 0 and grades 1 and 3. The trend seen pre-augmentation (i.e., more height loss and increased stress) changed.

### The von Mises stresses on fresh OVCFs before and after augmentation

**Fig. 9:**
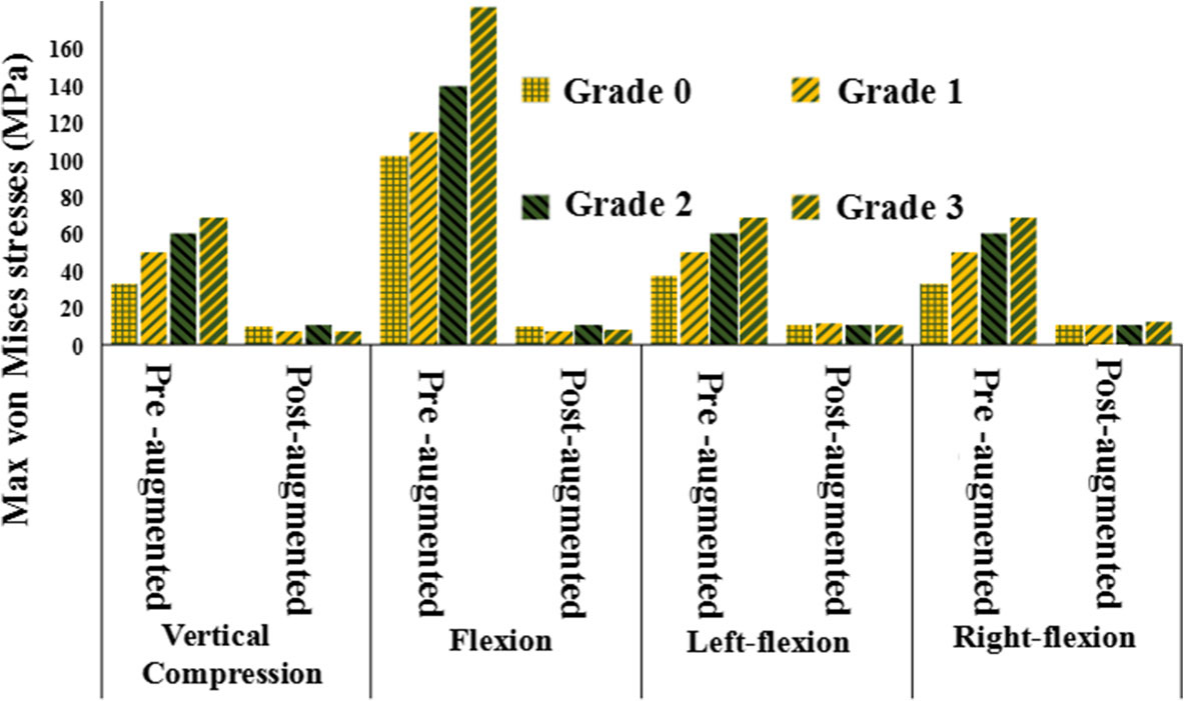
Comparison of the von Mises stresses before and after augmentation

A comparison of the von Mises stress in T12 OVCF models of grade 0–3 VHs before and after augmentation is shown in Fig. 9 and Table 2. All von Mises stresses were significantly decreased post-augmentation for different VHs. There were significant differences in preoperative and postoperative loads. The most obvious reduction and most significant change was observed for grade 3 VH, from 181.93 MPa to 7.9141 MPa under flexion load. The trend of changes of stress in various conditions was not so obvious postoperatively as it was preoperatively.

**Table 2:** The von Mises stresses (MPa) on the T12 vertebral body

	Vertical compression		Flexion		Left flexion		Right flexion	
	Pre	Post	Pre	Post	Pre	Post	Pre	Post
Grade 0	33.282	10.252	101.89	9.6296	37.54	11.08	33.159	10.433
Grade 1	49.84	7.6211	115.07	7.641	49.831	11.39	49.893	10.575
Grade 2	59.93	10.392	139.52	10.505	59.931	10.396	59.927	10.431
Grade 3	68.966	7.3754	181.93	7.9141	68.97	10.629	68.954	12.681

*Pre* pre-augmented, *post* post-augmented

## Discussion

OVCF is an important health issue in the aging population, and it is a common fragility fracture involving the collapse, compression, or wedging of a vertebral body, which may be accompanied by a lasting, painful, and disabling condition of the spine [30]. Owing to the increase in prevalence of osteoporosis and life expectancy of humans, the incidences of OVCFs are also increasing [31]. Although conservative therapy of OVCFs is performed routinely, it is difficult for patients to tolerate long-term bed rest. The traditional surgical treatment is accompanied by a similar disadvantage, which may cause various complications, such as pneumonia, urinary tract infection, bedsores, and deep venous thrombosis, especially among elderly patients [32]. Treatment of OVCFs needs to restore the VH. However, due to the effects of osteoporosis and stress, some patients do not experience restored VH at all after clinical treatment. Although PKP and PVP can relieve the clinical symptoms of patients with OVCFs through VA, the VH of these patients cannot be fully reduced [6, 7], and there are no acceptable clinical reduction criteria. The present study showed that the vertebral bodies can be bear similar stress after VA, as they did not have more height loss or bear more stress. This result is consistent with that of other clinical studies.

The severity of fractures and vertebral deformities can be classified by images of the spine using several methods. The semi-quantitative technique of Genant is one of the most common methods. However, this preoperative classification has only a small predictive power for postoperative reductions [3]. Due to the aging of patients and a low bone density, there are several difficulties in treating OVCFs through surgical methods, which may result in loose internal fixation, fracture recurrence, etc. However, VA techniques, including PVP and PKP, are efficacious surgical treatment methods for OVCFs [33], and they have been widely used to treat OVCFs to relieve back pain and correct deformity of the spine. PVP and PKP have resulted in significant pain relief and an increase in life expectancy, as well as improvement in spine function [34]. Studies have not found any significant differences between PVP and PKP in pain relief, mental health, and movement of the spine. Pain relief was the main outcomes in all research studies of PVP and PKP. As one of the criterion for evaluating the effect of fracture treatment, restoration of the bone position should be assessed using preoperative and postoperative images. PVP and PKP can achieve a significant increase in VH and a significant reduction in the kyphotic wedge angle [35]. For the treatment of OVCFs, VA techniques serve as minimally invasive and effective surgical methods, although the extent of VH restoration is not consistent postoperatively.

It has also been shown that VA cannot completely restore the VH by PVP and PKP based on a comparison of radiographs and CT images. In our research study, the clinical results of preoperative and postoperative images of semi-quantitative grading of VH were not significantly different, but pain relief was confirmed, although the operation could not completely restore the VH. Experimental biomechanical studies have shown that PVP or PKP increased stiffness and strength [36, 37]. Moreover, the increased stiffness of the augmented vertebral body led to an increase in the load of adjacent vertebral bodies [38], increased failure load of the vertebral body after augmentation [39], improved the degree of VH restoration after augmentation, and increased the risk of new fracture [40]. Additionally, the volume and distribution of biological materials, such as bone cement, used in surgery may have a significant effect in studies of the mechanical load of the models [41, 42]. The result of FEA showed that augmentation of bone cement can increase the risk of adjacent vertebral fractures [36]. FEA also demonstrated that an increased volume of bone cement can have a significant effect on the occurrence of subsequent vertebral fractures after augmentation [43]. The Von Mises law is a theory used to analyze the stress distribution on materials and models. According to this theory, the stress produced is called the von Mises stress and it has been recently used for evaluating the stress distribution on important areas of the virtual model [44]. These results of von Mises stress suggest that there are some important biomechanical phenomena outside the results of good clinical studies. However, these studies did not specifically study the biomechanical changes of different VHs after VA. In our study, although the preoperative and postoperative reductions of VH were different in grade 0–3 VH, VA significantly reduced the stress on the vertebral body under different loads and VHs.

In the current study, four different VHs in FEA models of OVCFs were established based on measurements from postoperative CT images. Since unilateral and bilateral percutaneous augmentation can provide excellent pain relief and improvement of life quality [4], unilateral bone cement injection models were used. Results of the FEA method provided the following biomechanical evidence: with clinical treatment, the VH cannot be fully restored after surgical treatment. In the four models, all biomechanical parameters, including the Poisson ratio and elastic modulus, were used according to previous literature [20–23]. Compared with other FEAs, we used four FEA models of different VHs. Results of FEA showed that the greater the loss of VH, the greater the stress on the vertebral body before vertebral reinforcement; additionally, vertebral stress of different VHs was significantly reduced.

The limitations of these FEA models must be considered. In this study, because the shapes of cement in the postoperative vertebral body were not exactly the same, we did try to use the real cement augmentation model for FEA. However, we found that we could not control the different effects of different cement forms on the experimental results. The use of a cylindrical cement model is easy for computer calculations and ensures the repeatability of the study [19]. This study’s findings do not reflect the complexities of real-life situations since several factors were not considered (e.g., the FEA models were too simplified; multiple vertebral fractures were excluded; and muscle force and the bone cement shape and location postoperatively were not considered). All these factors could have affected the von Mises stress observed before and after augmentation. Since the anatomical morphology of the four models was inconsistent, the stress of different vertebral bodies may also lead to subtle changes in stress trends despite the use of the same loading method. The result of the comparison of postoperative loads between the four grades of VHs may have been affected by this limitation; there was a significant difference between grades 0, 1, and 3 and grade 2, rather than no significant difference between all four grades. Therefore, the biomechanical changes in the clinical setting cannot be fully understood by virtue of this analysis. Based on the present study, a future biomechanical analysis should include FEA models that more accurately reflect the human condition.

## Conclusions

FEA confirmed that vertebral loads can be significantly reduced after augmentation, although the restoration of VH is different from grades 0 to 3 VH postoperatively. VA procedures have a significant effect on recovery of the biomechanical properties of the vertebral body with an OVCF. Lastly, there is no need for surgeons to pursue anatomic reduction in the clinical treatment of OVCFs with VA if the treatment goal is to only achieve the relief of symptoms.

## Abbreviations

3D: Three-dimensional
ALL: Anterior longitudinal ligament
CL: Capsular ligament
CT: Computed tomography
DICOM: Digital Imaging and Communications in Medicine
FEA: Finite element analysis
ISL: Interspinous ligament
LF: Ligamentum flavum
OVCF: Osteoporotic vertebral compression fracture
PKP: Percutaneous kyphoplasty
PLL: Posterior longitudinal ligament
PVP: Percutaneous vertebroplasty
SSL: Supraspinal ligament
VA: Vertebral augmentation
VAS: Visual analog scale
VH: Vertebral height
